# Impact of the Dopant Species on the Thermomechanical Material Properties of Thermoelectric Mg_2_Si_0.3_Sn_0.7_

**DOI:** 10.3390/ma15030779

**Published:** 2022-01-20

**Authors:** Gustavo Castillo-Hernández, Eckhard Müller, Johannes de Boor

**Affiliations:** 1 Institute of Materials Research, German Aerospace Center, 51170 Cologne, Germany; gustavo.castillo-hernandez@dlr.de (G.C.-H.); eckhard.mueller@dlr.de (E.M.); 2 Institute of Inorganic and Analytical Chemistry, Justus Liebig University Giessen, Heinrich-Buff-Ring 17, 35392 Giessen, Germany; 3 Institute of Technology for Nanostructures (NST), Faculty of Engineering, University of Duisburg-Essen, Bismarckstreet 81, 47057 Duisburg, Germany

**Keywords:** mechanical properties, thermoelectric, Mg_2_Si, Mg_2_Sn, thermal expansion

## Abstract

Thermoelectric generators are an excellent option for waste heat reuse. Materials for such devices have seen their thermoelectric properties improving constantly. The functioning of a generator, however, does not only depend on thermoelectric properties. Thermal and mechanical properties play a decisive role in the feasibility of any thermoelectric generator. To shed light on the properties exhibited by thermoelectric materials, we present the temperature dependent characterization of Young’s modulus and coefficient of thermal expansion for Mg_2_Si_0.3_Sn_0.7_. Comparing undoped to Bi-doped n-type and Li-doped p-type material, we investigated the influence of doping in the relevant temperature regime and found the influences to be minor, proving similar properties for n- and p-type. We found a Young’s modulus of 84 GPa for the p-type and 83 GPa for the n-type, similar to that of the undoped compound with 85 GPa. The thermal expansion coefficients of undoped, as well as n- and p-type were equally similar with values ranging from 16.5 to 17.5 × 10^−6^ 1/K. A phase analysis was performed to further compare the two materials, finding a similar phase distribution and microstructure. Finally, using the gathered data, estimations on the possible thermally induced stresses under a temperature difference are provided to evaluate the relevance of knowing temperature dependent thermal and mechanical properties.

## 1. Introduction

Thermoelectric generators (TEG) are solid state devices that can convert waste heat into usable electricity [1]. TEG have several advantages compared to other electrical power generation technologies in that they have no mobile parts and thus have low maintenance costs and high reliability and can function in the absence of light, in contrast to photovoltaic technology.

TEG can be manufactured from a wide range of materials, some of which are light and inexpensive [2,3]. The basic unit of such a TEG is a pair of doped semiconductors called legs, one n-type and the other p-type. Both legs are joined to a metallic connector usually denominated as the bridge. The legs are thus connected electrically in series and thermally in parallel [1,4], allowing to convert a fraction of the heat flowing through the legs into electricity. 

The legs are ranked according to the power generating capabilities they possess. This classification is summed up in the dimensionless figure of merit *zT*, which is defined as *zT* *=* *S*^2^*ρκ*^−^^1^*T*, where *S*, *ρ*, *κ*, and *T* represent the Seebeck coefficient, electrical resistivity, total thermal conductivity, and absolute temperature, respectively.

Among the materials that show good *zT* values, as well as other desirable properties like low density and cost, are the Mg_2_Si-Mg_2_Sn solid solutions. These materials have been thoroughly studied before, with *zT* values of 1.2–1.4 at 973 K for the n-type [5,6,7,8,9,10,11], while the p-type value is about 0.55 at the same temperature [12,13,14,15,16,17]. In conjunction with a density ranging from 1.99–3.5 g/cm^3^, the material system becomes a prime candidate for low-cost and non-toxic TEG technology development.

For TEG design, not only is the development of the thermoelectric properties important, but several other challenges need to be tackled as well. In particular, progress on contact technology and mechanical stability is also important. Contacting technology for Mg_2_Si-Mg_2_Sn has shown substantial progress as several candidate schemes have been evaluated [18,19,20,21,22] and their thermal stability assessed [23]. Mechanical properties have been studied, with our previous work detailing the temperature and composition dependent elastic behavior for the whole solid solution series [11,24,25,26].

Silicide-based TEG have traditionally been manufactured with an n-type Mg_2_(Si,Sn) and an higher manganese silicide (HMS) p-type leg because of the poor properties exhibited by p-type Mg_2_(Si,Sn) [25,27,28]. HMS has, on the other hand, quite different mechanical properties compared to Mg_2_(Si,Sn). In recent developments, however, both p-materials have achieved similar thermoelectric (TE) performance [12], and modules using only Mg_2_(Si,Sn) seem to be a realistic possibility now. Using n- and p-type legs from the same material class with similar compositions can be highly advantageous as the thermal and mechanical properties are expected to show similarity. This similarity is especially important since it has been proven that differences in the coefficient of thermal expansion (CTE) for the materials used in the legs may cause high thermally induced mechanical stresses, potentially damaging or destroying the module [29]. Moreover, the effect of damage caused by mechanical stress in modules, even if not destroying the module completely by a fracture, has been shown to decrease the device figure of merit to less than half the original value [20,30].

Having the same CTE for both leg materials is, however, not a guarantee that the module will have mechanical integrity, as other effects such as bridge or substrate expansion need to be considered. Previous work has been done on modeling the mechanical behavior of a Bi_2_Te_3_ module, where the said module employed legs that had the same CTE and Young´s modulus, but high stresses were found in the TEG module [31].

Since the thermal expansion will cause stresses even if the CTE of the TE materials is similar, it is important not to design a module only based on the CTE, but take into account Young´s modulus and Poisson’s rate of the materials as well.

Mechanical properties of materials with similar compositions and an identical microstructure are expected to be equally similar. However, doping species have been known to alter the mechanical response in some TE materials. Skutterudites, in particular, typically have a Young´s modulus of *E* > 140 GPa for n-type materials, while p-type materials rarely exhibit higher values than 130 GPa [32]. Within the same doping type, we see slight differences as well. P-type didymium (mixture of praseodymium and neodymium) filled material DD_0.86_Fe_4_Sb_12_ shows a Young’s modulus of 123 GPa, while the composition DD_0.68_Fe_3_CoSb_12_ reaches 127 GPa. DD_0.68_Fe_4_Sb_12_ has also been tested and shows a Young’s modulus of 105 GPa, but here it remains unclear if the difference is due to changes in composition or mainly due to a modified synthesis approach. 

Mg_2_X material belongs to space group $Fm\bar{3}m$ with Mg filling the 8*c* Wyckoff position and X the 4*a* position. X can be filled with Si and Sn to produce the Mg_2_(Si,Sn) solid solution. Doping for n-type is also done in this position. The typical n-type dopants Bi and Sb substitute X, as discussed e.g., in [33]. Bi-doped Mg_2_(Si,Sn) shows good thermoelectric properties [10,34,35] and thus, the effect of Bi on other properties has received more attention lately. The hardness in a Bi-doped Mg_2_Si material was reported to increase [26] from 327 Hv in undoped material to 475 Hv with an atomic dopant percentage of 2.5%. The authors of this work attribute the hardness increase to the substitution of Si by Bi in the materials crystal lattice. Note that the original work reports the change in composition as 0.0025 at%, which is very likely a typographical error. 

The lattice parameter of the Bi-doped cubic Mg_2_(Si,Sn) has been studied as well. Previous work details the effect of up to *x* = 4 at% Bi in a Mg_2_Si_0.35_Sn_0.65-*x*_Bi*_x_* material. In this case, the lattice parameter increased from 6.607 Å to 6.632 Å with no indication of a solubility limit [34]. The authors attribute the increase to Bi occupancy of Si, Sn place in the lattice.

As most previous studies have focused on the effect of Bi on the thermoelectric properties of said materials, little is reported about the CTE and the Young’s modulus, which are important for the stress formation in TEG modules in service. As high stresses may result in damages impairing the thermoelectric efficiency and finally may affect the structural integrity of the TEG, we performed the first ever characterization of CTE and Young’s modulus for a p-type Mg_2_(Si,Sn) material, in comparison with the n-type and undoped material. A discussion of the potential consequences for TEG development is also presented.

## 2. Materials and Methods

Mg_2_Si_0.3_Sn_0.7_ samples with different doping levels and species were synthesized using a mixed method described elsewhere [10]. The doping amount for n-type (3.5% Bi) and p-type (3% Li) was chosen according to previous work [10,12]; these compositions yield the best possible thermoelectric properties for the synthesis route. The low 0.75% Bi sample was chosen as initially, Bi segregation was deemed likely to happen and the effect of this was to be studied. However, as described later, no Bi-rich secondary phases were observed.

Precursor materials were Mg turnings (Merck, Darmstadt, Germany), Si chunks (<6 mm, ChemPur, Karlsruhe, Germany), Sn (<71 μm, Merck) with high purity > 99.5%, Li and Bi. A pellet was pressed from the powder in a direct current sinter press DSP 510 SE (Dr. Fritsch GmbH, Fellbach, Germany). The parameters of temperature ($T_{sinter}$) and pressure ($p_{sinter}$) used to sinter each sample are detailed in Table 1. Samples containing no Li were synthetized using extra Mg to account for losses in the process due to evaporation in the synthesis and pressing steps. These samples require, thus, extra time in the sintering step.

The pellets obtained had a diameter of 50 mm and a thickness of 3.5 mm. They were cut using a diamond disc saw (DISCO Corp., Tokyo, Japan) into pieces measuring (12 × 45 × 3.0) mm^3^ for the Young´s modulus measurement and (5 × 40 × 3.0) mm^3^ for the CTE measurement. Small semi-circular segments of the pellets were embedded in conductive resin, grinded with SiC paper, and polished with diamond suspension for microstructure analysis. 

The Impulse Excitation Technique (IET) was used to determine the Young’s modulus. Its measuring principle is based on the free vibration of a sample (bar or pellet) set on top of supports. It has been extensively described by other authors, as well as in our previous work [24,36]. Young´s modulus measurement was done using a device from Integrated Material Control Engineering NV (Genk, Belgium). High temperature characterization was done in air from RT until 673 K with a heating and cooling rate of 1 K/min, and a holding step of 60 min at maximum temperature was established. One data point was obtained every 30 s throughout the whole process. The cooling process can be controlled by the device down to 423 K; afterwards the cooling happens through natural convection. Two independent measurements were done per composition, the variation between them was lesser than the measurement precision and thus, this precision is reported.

The coefficient of thermal expansion was measured on a Bähr thermoanalysis dilatometer (Hüllhorst, Germany) in the temperature range of 300–720 K, using a sapphire calibration. The measurement was performed under vacuum (<1 × 10^−4^ bar) with a heating ramp of 1 K/min.

X-ray diffraction was used to identify the phases present. Such a measurement was performed on pieces of the obtained pellets utilizing a Bruker D8 advance diffractometer (Billerica, MA, USA) using Cu-Kα radiation (1.5406 Å) in the 2θ range 20°–80° with a step size of 0.01°. The Bragg equation was employed to estimate lattice parameters using the main diffraction peaks (111) and (220). Microstructure analysis was carried out using a Scanning Electron Microscope (SEM) Zeiss Ultra 55 SEM (Oberkochen, Germany) with a Zeiss QBSE detector, also equipped with an Oxford energy dispersive X-ray (EDX) detector (PentaFETx3) (Milpitas, CA, USA). The grain size was observed through SEM pictures and estimated using ImageJ on an average of 30 grains.

The electronic transport properties were measured utilizing an in-house developed facility utilizing a four-probe technique [37,38]. Density measurements were obtained using the Archimedes method in ethanol. 

## 3. Results

**Figure 1:**
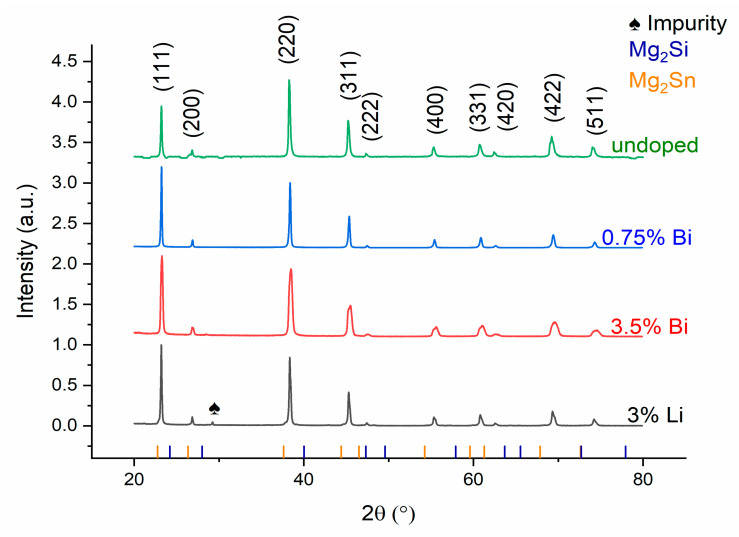
X-ray diffractograms of the samples studied.

XRD patterns shown in Figure 1 along with standard Mg_2_Si and Mg_2_Sn patterns confirm the presence of phases belonging to Mg_2_(Si,Sn) for the Li doped sample where there is also one unidentified impurity peak (~30°theta). The peak could be related to LiO_2_ or SiO_2_ but cannot be identified with certainty.

As can be seen from Table 2, we do not see a systematic change of the lattice parameter with a change in doping species or with an increasing Bi content. Previous work, in comparison, shows a systematic increase with increasing Bi substitution in the lattice [26,35,39,40] in the range of 0.01–0.03 Å, depending on the Bi amount. This apparent inconsistency could be related to the broadness of the peaks. The n-type with 3.5% Bi has broader XRD peaks compared to other compositions, possibly indicating the presence of several similar phases or compositional variations within on phase. An exemplary deconvolution into two different compositions, see Supplementary Information Figure S1, shows that the (220) peak is composed of 2 main components positioned at 2θ = 38.379° and 37.586° (Table S1 in Supplementary Information), which correspond to material having an *x* (Sn content) for Mg_2_Si_1−*x*_Sn*_x_* of 0.67 and 0.59, respectively. Our research focuses on upscaled material with a higher yield. It is thus not unexpected to find a range of compositions in such a big sample. 

The position and occupation fraction of the dopants can thus not be determined from the XRD pattern directly, but it is clear from the thermoelectric properties discussed later on that doping has been successful, i.e., Bi occupies the 4a positions, while Li tends to go to the 8c position as discussed e.g., in [41].

The grain size of all samples is comparable, which is most likely due to the similar preparation route. An example can be seen in Supplementary Information Figure S2.

Samples obtained using the same method and the same parameters have recently been shown to have state-of-the-art thermoelectric properties with *zT*_max_ = 1.3 at 773 K for the n-type [10]. The high carrier concentrations reported in these works $n\sim 10^{20}{\mathrm{cm}}^{-3}$ prove that the dopants have been incorporated and are active. The charge carrier density was estimated assuming a single parabolic band model and using the measured Seebeck coefficient as well as an effective mass of $m_{D}^{*}=1.43$ for the p-type material, while $m_{D}^{*}=2.5$ was used for the n-type and undoped materials [13,42]. The mobility (*µ*) was estimated using the equation ς=neµ where ς is the electrical conductivity, *n* is the charge carrier density, and *e* is the charge of an electron. Electronic transport properties of the samples are shown in Table 3

Our previous work has proven that the material shows a linear dependence of elastic moduli on *x* [24]; this work also provides evidence on the little difference in the mechanical properties we would expect for such small differences in composition. 

**Figure 2:**
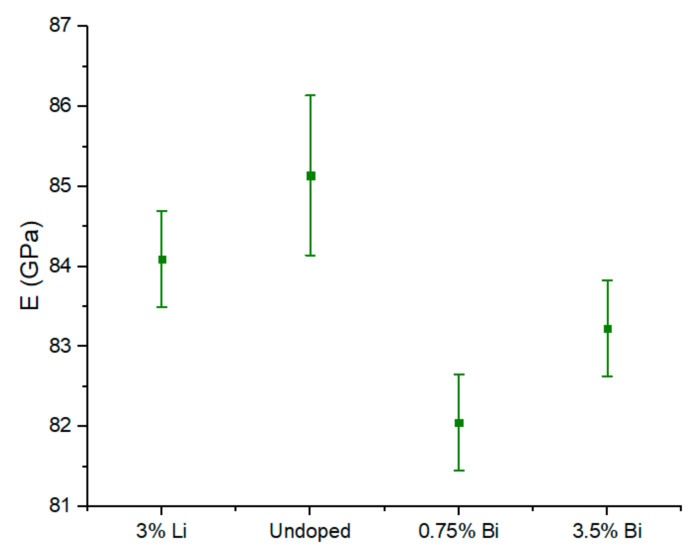
Room temperature Young’s modulus of the Mg_2_Si_0.3_Sn_0.7_ samples.

Previous work on Bi-doped Mg_2_(Si,Sn) shows that the lattice parameter keeps on increasing beyond 3 at.% Bi, however, the solubility limit can be assumed to be between 3 at.% and 4 at.% from the Seebeck and electrical conductivity values reported in [34]. It is therefore highly plausible that the range of Bi content within this study is within the solubility limit of Bi in Mg_2_(Si,Sn). Comparison with the work of Nieroda et al. [16] furthermore indicates that the Li-content in our sample is well below the solubility limit. The room temperature mechanical properties exhibited by the samples are shown in Figure 2. Samples without Bi have a slightly higher Young’s modulus.

**Figure 3:**
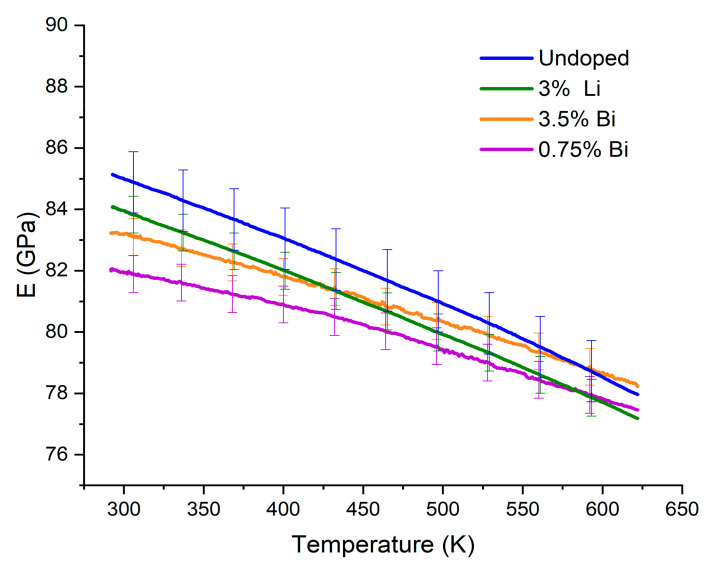
High temperature Young’s modulus of the Mg_2_Si_0.3_Sn_0.7_ samples.

All materials studied in this work exhibit a general similarity in mechanical properties. This behavior is presumably due to the overall similarity in composition but the minor differences in composition lead to some small differences in high temperature Young’s modulus. Such differences can be seen in Figure 3. Undoped and p-type Li-doped samples show the same slope of temperature dependency and a small difference in absolute values.

We can thus prove that at relevant application temperatures, undoped and both n- and p-type doped Mg_2_Si_0.3_Sn_0.7_ show similar absolute values and similar temperature dependence of the Young’s moduli. The minor amount of impurity found in the 3% Li doped sample did not affect the room temperature values of the said material significantly.

In our previous work, we provided information about the Poisson ratio and argued that the value could be considered as constant in the temperature region studied by this work [24]. The room temperature measurement results for the shear modulus *G* are shown in Table 4, as well as the values for Poisson’s ratio estimated using the equation $\nu =\frac{E}{2G}-1$.

**Figure 4:**
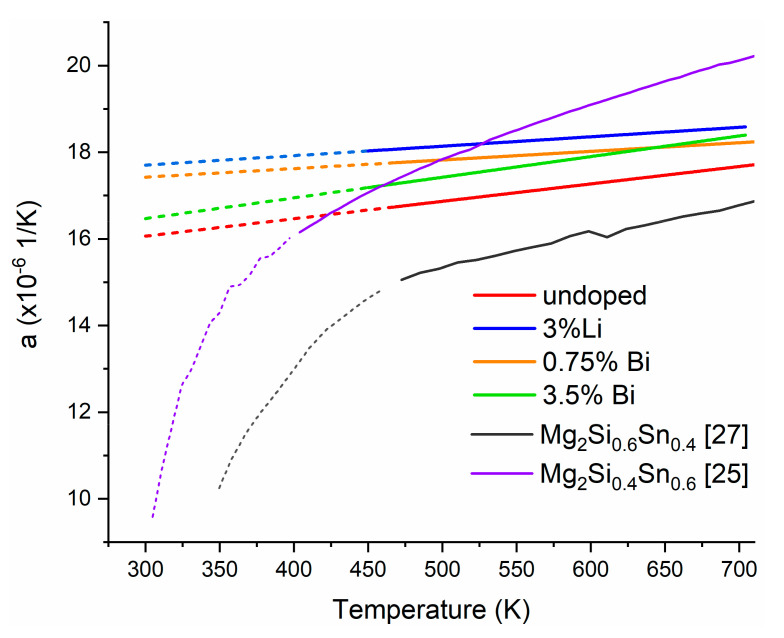
CTE between room temperature and 700 K for all samples of this study and selected literature results. Values for comparison are adapted from [25,27] in purple and black, respectively. Full lines depict linear behavior range, dashed lines show the extrapolation to room temperature of our measurements, and dotted lines indicate the strongly non-linear region in the reference data.

The coefficient of thermal expansion (*a*) data as a function of temperature is given in Figure 4 and shows two distinct parts: A strongly non-linear behavior from room temperature to ~400 K, which according to literature, stems from thermal inertia originated from internal stress [43], followed by an almost perfectly linear correlation between temperature and *a*. The raw data and extrapolation process are shown in the Supplementary Information Figure S3.

Previous first principles calculations performed by Ganeshan et al. on the binaries Mg_2_X (X = Si, Sn) predict a linear behavior of both the cell volume and CTE above room temperature [44]. These values were derived from the vibrational free energy per atom calculated from the phonon density of states [44]. Assuming the same linear behavior for our material and taking the values of the second region as well, the CTE values for low temperatures were derived by extrapolating the linear function *a*(*T*) from the high temperature regime between 450 and 700 K, and these values are shown in Figure 4. CTE values for the samples range between room temperature and 700 K. The linear function *a*(*T*) will be used in subsequent estimations, however an example of raw data for elongation and CTE is available in Supplementary Information Figure S4.

The *a* values from the Mg_2_Si_0.4_Sn_0.6_ are consistent with our data. The work done on this material reports a mean value of 17 × 10^−6^ 1/K [25]. The temperature dependence of the CTE in this work was obtained by dividing the reported elongation value by the temperature at which the data was obtained.

In the case of the Mg_2_Si_0.6_Sn_0.4_, the slope is similar to what we report, albeit with lower absolute values. This can be explained by the increased Si content in the material, as it is known that binary Mg_2_Si has an *a* value of 14 × 10^−6^ 1/K [45], and therefore a material with a higher Si content would be expected to have a lower CTE, closer to the binary.

## 4. Discussion

Previous work has detailed the effect of Bi doping on binary Mg_2_Si and the Mg_2_Si-Mg_2_Sn solid solutions; the solubility limit of Bi in the material, as well as its effects on the thermoelectric properties were described in [34,35,39,46], while different mechanical properties of the material with different Bi concentrations were detailed in [26]. 

The authors of some of the previous works have reported phases outside the Mg_2_Si-Mg_2_Sn solid solution like MgO, Mg_3_Bi_2_, and SiO_2_ in the samples, some of which increase systematically as the Bi content increases.

These phases, as well as regions with different *x* Sn content could affect the mechanical response of the material [47]. Our XRD patterns show a very minor phase not belonging to the Mg_2_Si-Mg_2_Sn material system, which seems to have no effect on the microstructure or mechanical properties measured.

However, the width of the peaks in the sample with 3.5% Bi is larger than that of the others. The compositions found through deconvolution of the peak have a *x* Sn content difference below 0.1, which, according to previous work on the dependence of the Young’s modulus on the Bi content, would yield a difference in the Young’s modulus of < 3 GPa. 

Previous studies have described phase formation from the elements into Mg_2_(Si,Sn) under milling, where, in the presence of both Si and Sn, Mg_2_Sn tends to form first and then Si from brittle elemental debris slowly diffuses into the Mg_2_(Si,Sn) matrix [48]. This process might be influenced by the miscibility gap in the Mg_2_Si-Mg_2_Sn quasibinary system which is controversially discussed [49,50]. However, as discussed in [48], this could be the reason for the observed sharp contrast between regions of different Si content.

Longer sintering processes, studied in [44], were found to reduce the size and number of the Si-rich areas. However, a short process is technologically desirable, moreover the interfaces related to these inclusions are also known to act as phonon scatterers, reducing the thermal conductivity [13]. Inclusions with different mechanical properties also influence the mechanical properties. They are an intrinsic way to strengthen a material [51] and thus, a small number of areas with different *x* Sn content can be beneficial for the overall performance of the TE material. 

The phase quantification was done following the procedure described in [52] on the four investigated materials. As detailed in the original publication, the Mg content is taken as constant (66.6 at%) and Si and Sn account for the difference to unity. The only degree of freedom is thus, the Si:Sn ratio.

The gray value obtained from the backscatter electron image was related to a composition measured by EDX, this relationship was then used to estimate the composition in the complete area observed through SEM. 

**Figure 5:**
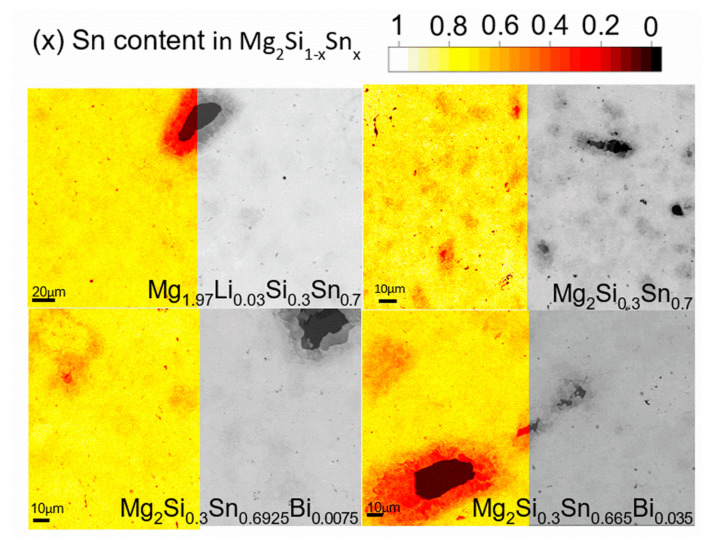
SEM images of the four studied materials; partly overlaid with color-coded plots visualizing the Sn concentration. For undoped material the same SEM picture shown in [24] was used as a base for the analysis.

Figure 5 displays SEM images of the four investigated materials. On half of each image, the Sn concentration is displayed as a color-coded overlay. 

From the compositional distribution estimated and shown in Figure 5, the mean composition was determined by plotting a histogram of the individual point compositions and fitting a Gaussian peak to the distribution (see Supplementary Information Figure S5). The peak center and full width at half maximum (FWHM) were taken as mean phase composition and its variation respectively. The results are shown in Table 5 whereas the graphs corresponding to the fitting can be found in the Supplementary Information.

It can be seen that all samples are located around *x* = 0.7 for Mg_2_Si_1−*x*_Sn*_x_*, with similar variation in their composition. This is partially due to the similarity in the preparation method that is melting followed by crushing the ingot in a high energy ball mill. As the mean and distribution width values are estimated from the grey value of the BSE micrographs, the method tends to overestimate the variation in composition. 

The composition histogram calculated through the phase quantification was used as a simple base for the calculation of effective mechanical properties, for the following estimations, the whole histogram (SI) was used and we can define $n_{i}$ as the fraction of the total material that has a specific i Sn content.

We used the linear equation to predict elastic moduli that we proposed in a previous work: $E\left(T,x\right)=E_{r}+bT+cx$ where $E_{r}=116.5\;GPa$, $b=-0.0234\;GPaK^{-1}$, and c=−32.032 GPa. Since the calculations are done at room temperature, we set T=300K [24].

Values for *x* Sn content were taken from the compositional percentages calculated (see SI) and thus the elastic modulus characteristic to that specific composition $E_{i}$ is defined, we find that both the Voigt ($\bar{E}=\underset{i}{\sum }n_{i}E_{i})$ and Reuss E¯=∑iniEi−1 approximations yield a theoretical elastic modulus of 87 ± 2 GPa for all samples. This is in line with the measured value for the undoped material of 85.14 GPa. The difference to the actual values of the doped samples (which are between 4% and 6% larger) stems probably from using the relation of *E*(*x*) for the undoped material, obtained in our previous work whereas the slightly overestimated variation in composition is caused by the quantification method. This variation in turn is within the same range as the precision of the measurement presented.

Mechanical properties are heavily influenced by the nature of the bonding between atoms and hence the composition, in this case the Si:Sn ratio, is known to have an effect on the Young’s modulus [24,53].

Similar changes might be expected due to doping, however on a smaller scale due to a smaller change of composition. Such a change is material specific and not clear a priori. Having established that our material is secondary-phase free and confirmed through local composition estimation that the Si:Sn ratio is similar, we can prove that both n- and p-type materials behave similarly at application temperatures. Moreover, the drastic hardness differences reported for Mg_2_Si in [26] are most likely linked to secondary phases, not the intrinsic material properties.

In an application, the thermoelectric materials will be assembled in a generator, being soldered or otherwise joined to metallic contact bridges fixed to insulating, often ceramic substrates. In this configuration and with a variation of temperature, stresses will arise due to the different expansion of TE material and bridge. 

The magnitude of the stresses occurring in the TEG depend on the design, the operating conditions of the TEG, and the thermal and mechanical properties of the TEG materials. For stationary conditions, the material parameters CTE, Young’s modulus and Poisson’s ratio are sufficient to calculate the stresses. 

For example, the maximum stress *σ* in a fully restrained material sample, which has been subjected to a temperature change, is defined by Equation (1) [54].

(1) $$\sigma =\frac{Ea\left(T_{0}-T_{1}\right)}{1-\nu }$$

where $T_{0}$ and $T_{1}$ are the temperatures before and after heating the material sample, ν is the Poisson’s ratio, and *a* is the coefficient of thermal expansion.

If we analyze the case where we are at the threshold of failure, where the fracture tensile stress $\sigma_{u}$ is reached, and using

Equation (1), we can identify the maximum sudden temperature change *T*_0_ − *T*_1_ that a material can withstand [54,55]. This parameter is also called the thermal shock resistance *R*, which is defined by Equation (2) [54,56]:

(2) $$T_{0}-T_{1}=\frac{\sigma_{u}\left(1-\nu \right)}{Ea}=R.$$

This equation is valid when the surface temperature of the material sample changes instantaneously.

If the heat transfer is not instant but kept at a constant rate, then the speed at which the heat flows from the core to the outer layer in a cylinder-shaped sample, and from there to the ambient, will also play a decisive role in the stress distribution. In this case, a second thermal resistance parameter, *R*′, is employed, whose governing equation is:

(3) $$R’=\frac{\kappa \sigma_{u}\left(1-\nu \right)}{Ea}.$$

In both cases, the product *E·a* is an important parameter to characterize a material subjected to temperature differences. 

**Figure 6:**
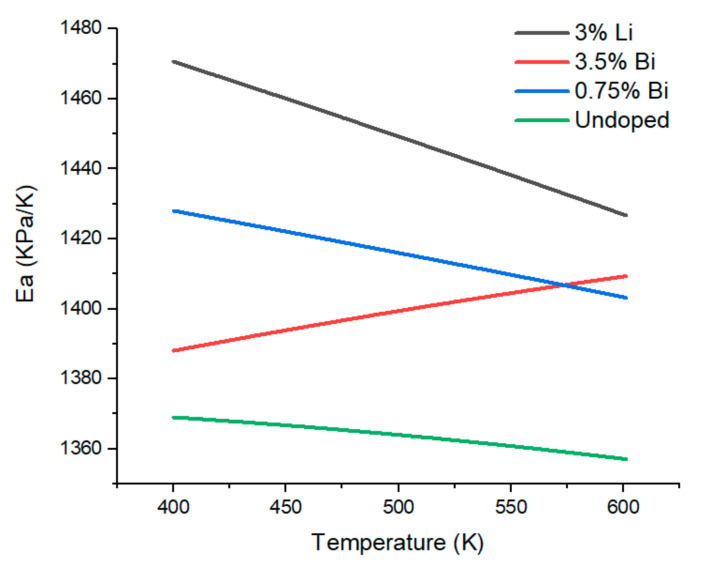
Temperature dependence of (*E·a*), the product of Young’s modulus and CTE.

We, therefore, used a linear fit for the thermal dependence of *E* (as shown in Figure 3) and *a* (as shown in Figure 4) and plotted the behavior of the product *E·a* in the target application temperature range 400–620 K as shown in Figure 6. Note that not $E\cdot a\;/\left(1-\nu \right)$ but *E·a* is plotted, the order of the curves is slightly modified, see Figure S6 in Supplementary Information. However, it is still the p-type that develops the highest stress among the optimized TE materials.

The temperature dependence of *E·a* in the thermoelectric optimized materials exhibits a convergent behavior. Both n- (3.5% Bi) and p-type (3% Li) exhibit a very similar value at application temperature and thus, are expected to develop similar thermally induced stresses.

Other thermoelectric material systems have comparable *E·a* values for the temperature range between 400 and 600 K: Ba_8_Ga_16_Ge_30_ shows a value of 1462 kPa/K, while Sr_8_Ga_16_Ge_30_ has a value of 1198 kPa/K. Tellurides show a lower value, with 1148 and 674 for PbTe and Bi_2_Te_3_, respectively [55]. A more direct comparison can be done to Skutterudites, the mechanical properties of these materials are also well known [32,57] and thus their *E·a* values can be estimated. Such values range from 1129 kPa/K for DD_0.76_Fe_3.4_Ni_0.6_Sb_12_ to >1700 kPa/K for CoSb_3_. Silicide-based TEG have thus an *E·a* product comparable to skutterudites, with the added advantage of a lower density and toxicity.

Using the previously detailed parameters, it is also possible to predict the thermally induced stress the material of a single leg of a thermoelectric module could have if it would be confined in length and heated from a homogenous temperature *T*_0_ to higher homogenous temperature *T*_1_. Using Equation (1) and the data presented in this work, we estimated the theoretical stress the leg would undergo for *T*_1_ values between 400 and 600 K if *T*_0_ is 325 K.

**Figure 7:**
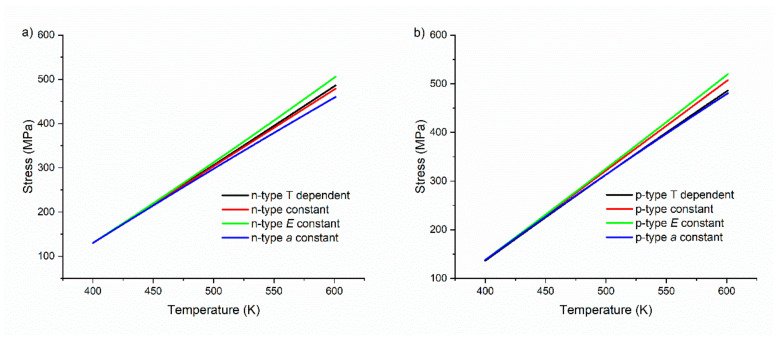
Comparison of thermal stress with both *E* and *a* as temperature dependent variables (black), both as constant with RT values (red), only *E* as constant (green) and only CTE as constant (blue) in material containing (**a**) 3.5% Bi and (**b**) 3% Li.

To visualize the effect of using temperature dependent data, this is compared to the hypothetical stress when the room temperature values of *E* and/or *a* are employed instead of the temperature dependent data, see Figure 7. 

As can be seen from both graphs, the values estimated for thermal stress are at a maximum when the Young’s modulus is considered constant. These values ignore the reducing of E with increasing temperature. The temperature dependence of the CTE has a small influence on the thermal stress as $\frac{da}{dT}\approx 10^{-9}\;K^{-2}$. 

By using the temperature dependent elastic modulus, however, the difference in stress at the temperatures studied is close to 10% in comparison to the use of room temperature values. 

These thermal stress values, however, only depict the effect of thermal expansion of a single leg and can be taken as an indication for the relevance of *T*-dependent mechanical properties. For a complete picture, it is necessary to consider, additionally, the effects of the expansion in the bridge and substrate. 

## 5. Conclusions

We presented the temperature dependent elastic properties exhibited by Mg_1.97_Li_0.03_Si_0.3_Sn_0.7_ and Mg_2_Si_0.3_Sn_0.665_Bi_0.035_ and compared it to undoped and low doped n-type material Mg_2.06_Si_0.3_Sn_0.6925_Bi_0.0075_. We observed a similarity between these values with a relative difference to the values of the undoped material of less than 4% at room temperature. The Young’s modulus is for all materials decreasing with an increasing temperature. Microstructural analysis shows that local fluctuation in Si:Sn observed for all samples does not affect their mechanical properties strongly. Furthermore, they can be predicted with good accuracy using the linear equation proposed and the composition range estimated through SEM pictures. 

The CTE values for both of these materials were measured. They all share similar values with differences between the n- and p-type being 6% at operating temperature.

The comparison between analytic stress estimation using room temperature measurements and temperature dependent data shows a ~10% difference at *T*_0_ = 325 K and *T*_1_ = 600 K due to the overestimation of the Young’s modulus in the constant data estimation, emphasizing the need for temperature dependent measurements if high accuracy is required.

We found that the difference in elastic moduli behavior in Mg_1.97_Li_0.03_Si_0.3_Sn_0.7_ and Mg_2_Si_0.3_Sn_0.665_Bi_0.035_ is partially accounted for with the difference in CTE, as the thermal stress developed by the legs is very similar. The similarity of both Young’s modulus and CTE in n- and p-type further confirms the viability of using Mg_2_Si_0.3_Sn_0.7_ for TEG development.

The data presented in this work expands the knowledge of mechanical behavior in TE materials, indispensable for developing a functional TEG with long life expectancy. The information is, however, not complete as the fracture stress of the materials is yet to be measured, as are the fatigue limits.

## Figures and Tables

**Table 1:** Composition and sintering time for the employed Mg_2_Si_0.3_Sn_0.7_ samples. We furthermore employed $T_{sinter}=973\;K$ and $p_{sinter}=66\;\mathrm{MPa}$.

Nominal Composition	Time (min)
Mg_1.97_Li_0.03_Si_0.3_Sn_0.7_	10
Mg_2.06_Si_0.3_Sn_0.7_	10
Mg_2.06_Si_0.3_Sn_0.6925_Bi_0.0075_	20
Mg_2.06_Si_0.3_Sn_0.665_Bi_0.035_	20

**Table 2:** Summary of structural properties for the Mg_2_Si_0.3_Sn_0.7_ samples.

Composition	Density (g/cm^3^)	Lattice Parameter (Å)	Grain Size (µm)
Mg_1.97_Li_0.03_Si_0.3_Sn_0.7_	3.10 ± 0.01	6.61 ± 0.01	7 ± 3
Mg_2.06_Si_0.3_Sn_0.7_	3.11 ± 0.01	6.63 ± 0.01	7 ± 3
Mg_2.06_Si_0.3_Sn_0.6925_Bi_0.0075_	3.09 ± 0.01	6.62 ± 0.01	6 ± 2
Mg_2.06_Si_0.3_Sn_0.665_Bi_0.035_	3.11 ± 0.01	6.61 ± 0.01	5 ± 3

**Table 3:** Electronic transport properties shown by the Mg_2_Si_0.3_Sn_0.7_ samples at 25 °C.

Composition	Seebeck (µV/K)	Electrical Conductivity (S/cm)	n (cm^−3^)	Mobility (cm^2^/Vs)
Mg_1.97_Li_0.03_Si_0.3_Sn_0.7_	101	644	1.7 × 10^20^	24
Mg_2.06_Si_0.3_Sn_0.7_	−453	29	3.7 × 10^18^	50
Mg_2.06_Si_0.3_Sn_0.6925_Bi_0.0075_	−157	1178	1.4 × 10^20^	53
Mg_2.06_Si_0.3_Sn_0.665_Bi_0.035_	−114	2138	2.8 × 10^20^	48

**Table 4:** Room temperature shear modulus and Poisson’s ratio.

Composition	Shear Modulus (GPa)	Poisson Ratio
Mg_1.97_Li_0.03_Si_0.3_Sn_0.7_	35.2 ± 0.3	0.193 ± 0.002
Mg_2.06_Si_0.3_Sn_0.7_	35.7 ± 0.3	0.191 ± 0.002
Mg_2.06_Si_0.3_Sn_0.6925_Bi_0.0075_	32.7 ± 0.3	0.217 ± 0.003
Mg_2.06_Si_0.3_Sn_0.665_Bi_0.035_	34.6 ± 0.3	0.209 ± 0.002

**Table 5:** Mean phase composition as calculated from the grey values from the SEM images by backscattered electrons for Mg_2_Si_1−x_Sn_x_.

Sample	Sn Content *x* and FWHM
Mg_1.97_Li_0.03_Si_0.3_Sn_0.7_	0.72 ± 0.11
Mg_2.06_Si_0.3_Sn_0.7_	0.73 ± 0.21
Mg_2.06_Si_0.3_Sn_0.6925_Bi_0.0075_	0.74 ± 0.15
Mg_2.06_Si_0.3_Sn_0.665_Bi_0.035_	0.69 ± 0.13

## Data Availability

The data presented in this study are available on request from the corresponding authors.

## Supplementary Materials

The following supporting information can be downloaded at: https://www.mdpi.com/article/10.3390/ma15030779/s1, Figure S1: Exemplary deconvolution of the (220) peak exhibited by the Mg_2.06_Si_0.3_Sn_0.665_Bi_0.035_ sample; Table S1: Fitted peaks for the XRD spectrum belonging to the sample doped with 3.5% Bi with peaks near the 38° mark highlighted; Figure S2: SEM backscatter image of the Mg_2.06_Si_0.3_Sn_0.665_Bi_0.035_ sample with markings for some grains used to estimate average grain size; Figure S3: (a) Coefficient of thermal expansion for an undoped Mg_2_Si_0.3_Sn_0.7_ after calibration showing the linear and non-linear regimes. (b) fitting and extrapolation done on the same data; Figure S4: (a) Raw data corresponding to elongation and (b) raw data corresponding to CTE, the CTE values were obtained by dividing elongation by temperature; Figure S5: Histograms for local composition quantification showing the mean composition and distribution; Figure S6: Sensitivity to thermal stress in all materials studied.
