# Supplementary material for: Impact of the Dopant Species on the Thermomechanical Material Properties of Thermoelectric Mg2Si0.3Sn0.7
Source: Materials (Basel). 2022 Jan 20;15(3):779. doi: 10.3390/ma15030779 (PMC8836357; doi:10.3390/ma15030779)
Supplement: Supplementary file 1 [file materials-15-00779-s001.zip › materials-1531544-supplementary.pdf]

Support Information

Impact of the Dopant Species on the Thermomechanical Material Properties of Thermoelectric $Mg_2Si_{0.3}Sn_{0.7}$

Gustavo Castillo-Hernández, Eckhard Müller and Johannes de Boor.

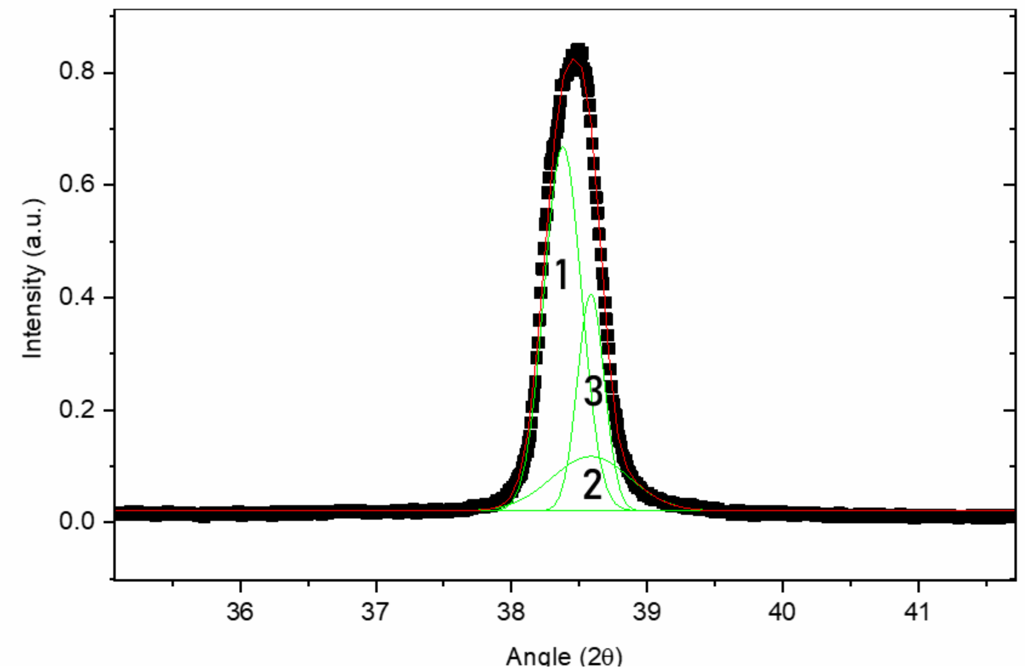

Figure S1. Exemplary deconvolution of the (220) peak exhibited by the $Mg_{2.06}Si_{0.3}Sn_{0.665}Bi_{0.035}$ sample.

Table S1. Fitted peaks for the XRD spectrum belonging to the sample doped with 3.5% Bi with peaks near the 38° mark highlighted.

Peak Index	Peak Type	FWHM	Max Height	Angle position
1	Gaussian	0.32442	0.64763	38.37889
2	Gaussian	0.68055	0.09691	38.58589
3	Gaussian	0.23375	0.38569	38.58592

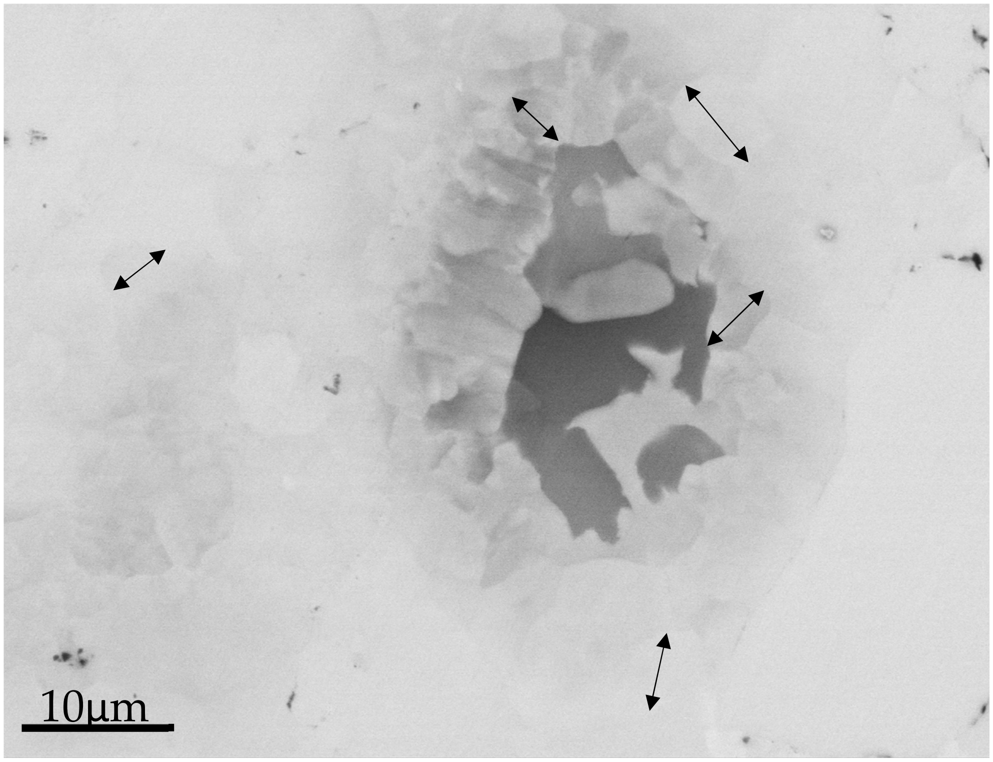

Figure S2. SEM backscatter image of the $Mg_{2.06}Si_{0.3}Sn_{0.665}Bi_{0.035}$ sample with markings for some grains used to estimate average grain size.

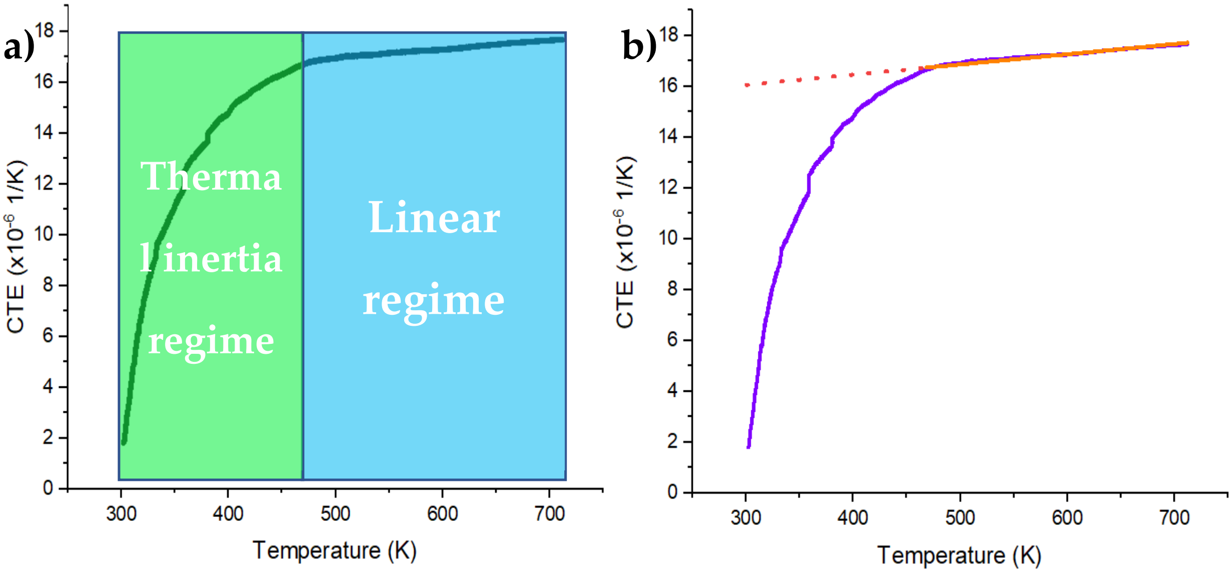

Figure S3. (**a**) Coefficient of thermal expansion for an undoped $Mg_2Si_{0.3}Sn_{0.7}$ after calibration showing the linear and non-linear regimes. (**b**) fitting and extrapolation done on the same data.

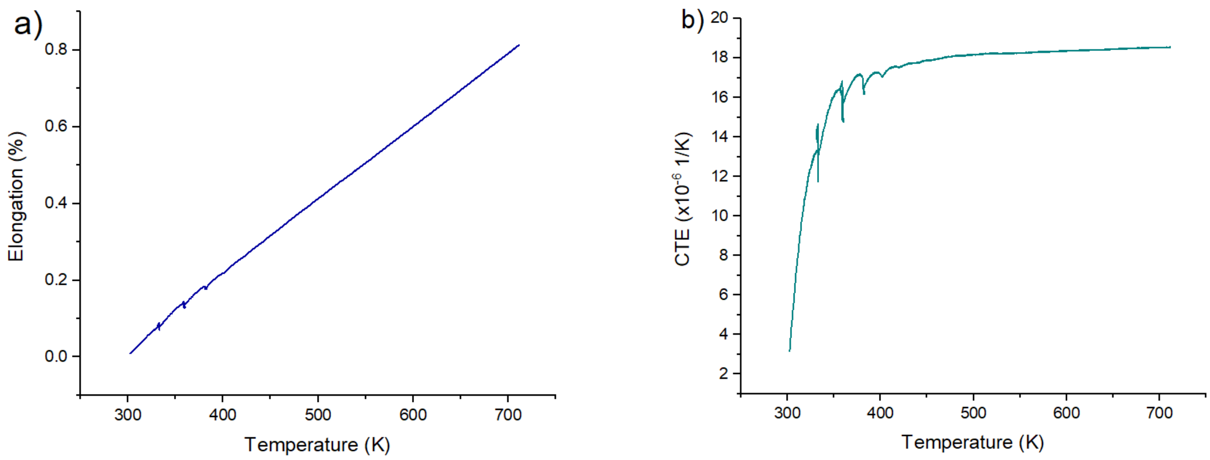

Figure S4. (a) Raw data corresponding to elongation and (**b**) raw data corresponding to CTE, the CTE values were obtained by dividing elongation by temperature.

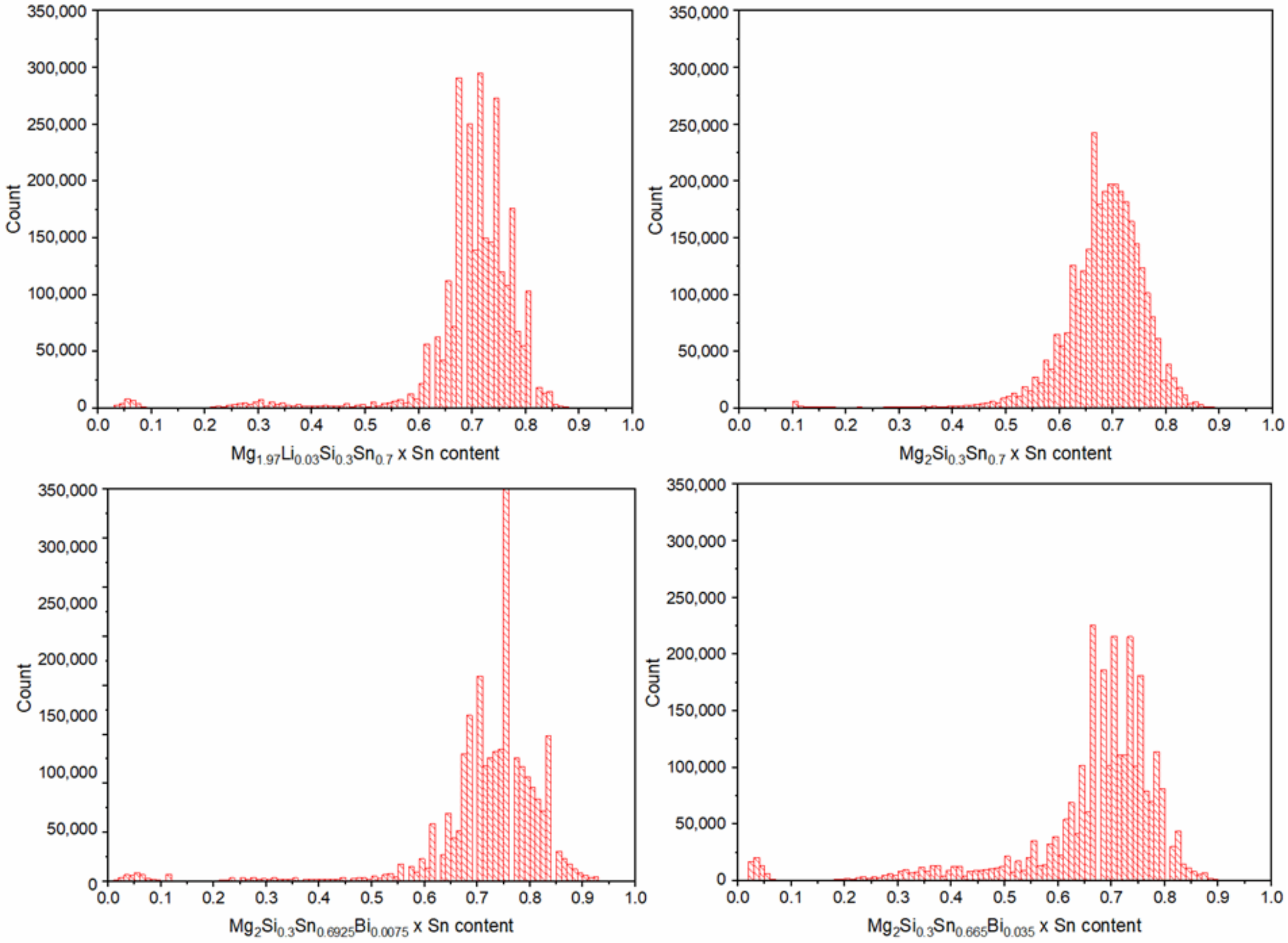

Figure S5. Histograms for local composition quantification showing the mean composition and distribution.

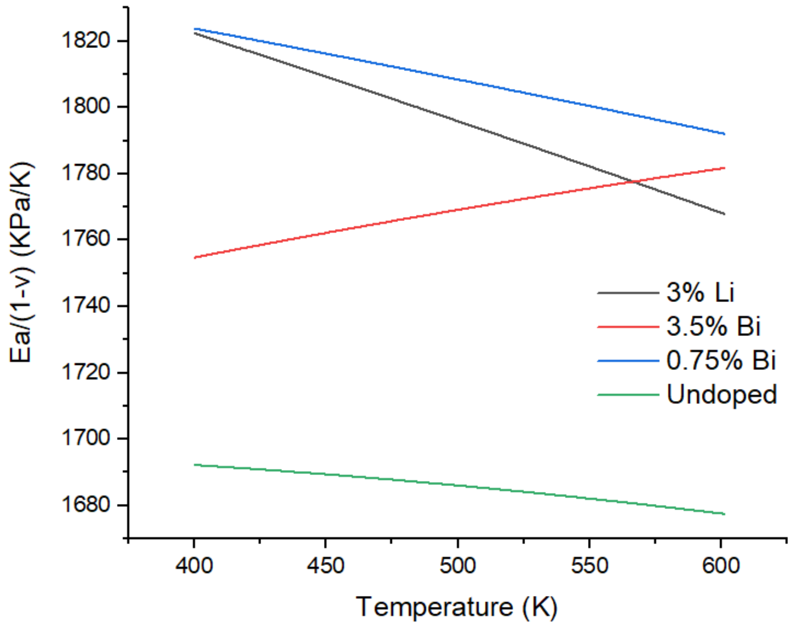

Figure S6. Sensitivity to thermal stress in all materials studied.
